# Inter- and intra-rater reliability of two aquatic safety skill assessment tools

**DOI:** 10.1016/j.jsampl.2025.100122

**Published:** 2025-11-25

**Authors:** N. Nyitrai, C. James, M. Brunton, S. Edwards

**Affiliations:** a The University of Newcastle, College of Health, Medicine and Wellbeing, Ourimbah NSW, Australia; b The University of Sydney, Sydney School of Health Sciences, Camperdown NSW, Australia; c School of Exercise Sciences, Australian Catholic University, Strathfield NSW, Australia

**Keywords:** Learn to swim, Assessment, Reliability

## Abstract

**Background:**

Learning to swim is recommended as an important layer of protection in drowning prevention. However, identifying what aquatic skill(s) are essential, and the absence of a gold or industry standard makes establishing the reliability of learn to swim assessment difficult.

**Methods:**

Five aquatic skills aligned with water safety and survival, from the Australian Water Safety Council's 2016 benchmark, were included in a cross-sectional study designed to test the reliability of two assessment tools: 1. competent/not yet competent and 2. RAEE (Refuse, Assisted, Effective, Efficient) assessment tool. Twelve participants (raters) from a single Gold Level AUSTSWIM recognised swim centre completed the assessment across three sessions and inter- (weighted kappa) and intra-rater (Chi squares) reliability was calculated.

**Results:**

There was limited/poor inter and intra rater reliability for both assessment methods and this increased across sessions for three of the five chosen skills (crouch dive, sidestroke and compact jump). RAEE assessment tool demonstrated lower inter- and intra-rater reliability (poor to fair) when compared to use of the C/NYC assessment method (fair to moderate) across five water safety skills.

**Conclusion:**

Regardless of the assessment approach taken, both inter and intra – rater reliability was limited when assessing water safety skills. A lack of consensus was found relating to proficiency in performance and raters lacked a clear understanding of the complexities involved in assessment, including an established foundation of what proficient motor skills performance looks like.

**Key Points**

• Community-based education of school aged children in basic swimming, water safety and self-rescue skills is a critical layer in drowning prevention.
• This study showed that irrespective of the assessment approach taken, both the inter and intra – rater reliability was limited when assessing water safety skills from the AWSC's 2016 benchmark level of swimming and water safety.
• A general lack of consensus among participants relating to what constitutes proficiency in performing aquatic skills, particularly motor skills related to water safety, as it was evident there was a clear lack of understanding of the complexities involved in assessment and what it looks like to perform each of these motor skills well.
• Taking measures to improve inter- and intra-rater reliability within the aquatics industry could improve the capacity to accurately measure the impact of swimming and water safety instruction as a drowning prevention tool.

## 1 Introduction

Drowning is recognised as a leading global public health issue with an estimated there to be 360,000 annual fatalities [1,2]. World Health Organisation state the best strategy to reduce the drowning toll would involve community-based initiatives, effective policy and legislation and further research, to collectively provide ‘layers of protection’ [1]. A critical layer in drowning prevention suggest is community-based education of school aged children in basic swimming and water safety skills [1,2]. There is no consensus about what constitutes the essential aquatic skills, knowledge and understanding, and how these can be reliably measured [[3], [4], [5]]. Nor is there currently a ‘gold’ standardised assessment tool used by the aquatics industry within Australia, which complicates the process of establishing reliability for any tools that may be developed.

Evaluating the effectiveness of learn-to-swim programs in developing the skills required to aid drowning prevention efforts in the absence of an appropriate standard, requires a reference point [6]. For example, a common reference point for swimming are the techniques used by adult elite athletes, encompassing four competitive strokes [7]. The Australian Water Safety Council's (AWSC) National Swimming and Water Safety Framework also provides a reference for the age appropriate benchmark levels of basic swimming, water safety and self-rescue [8]. For children leaving primary school (∼12–13 years), the Royal Life Saving's Swim and Survive Active Award 4 (RLSSA) or its equivalent, which was identified as an achievable level for 100 ​% of Australian children [9].

Reports such as the Royal Life Saving's annual national drowning reports and others investigating the state of Learn to Swim, have identified that only an estimated 40 ​% of students are meeting the benchmark level of swimming [[10], [11], [12], [13]]. This was attributed to a range of issues such as socio-economic, COVID 19 and teacher shortages, but notably the differences in the skills being taught across different learn-to-swim programs, varying levels of awareness of the benchmark and/or benefits of meeting the benchmark amongst stakeholders [11,[13], [14], [15], [16]]. The use of the generic term learn-to-swim across the various stakeholders leaves open to interpretation of what the skill of ‘swimming’ is. This has created uncertainty about the identification of what ‘foundational’ aquatic skills reflect the broad notion of ‘swimming’. When different skills are being taught and assessed in different programs the reliability of assessments and associated communication surrounding the attainment and/or retention of aquatic skills in the community will be compromised [3,17,18]. The difficulty in producing reliable assessment of aquatic skills is acknowledged within the aquatics industry [9,11].

Reliably quantifying motor skill performance, involves conducting specific tests and evaluating performance using established measurement tools to determine whether the objectives of the task are being met [6]. To be described as ‘competent’ in a motor skill in an outcome-based assessment, implies a practical demonstration of a specific skill and/or understanding by an individual that matches the criteria associated with the skill [6]. In the absence of a formal assessment process in learn-to-swim, Competent or Not Yet Competent (C/NYC*)* in the performance of a skill has become the defacto assessment method used by AUSTSWIM teachers of swimming and water safety [19]. Due to the open interpretation of ‘swimming’, and variety of learn-to-swim programs with differences in teaching philosophy, there is currently no process for communicating what standards, criteria or environment is being used by instructors for assessment and therefore, what the assessment outcome of ‘competent’ represents [18,19]. While specific aquatic assessment tools have been developed (particularly for people with a disability or pre-school children), there is currently no recognised assessment tool used in main stream (i.e. school age) learn-to-swim programs [17,18,20,21].

The limitations of the C/NYC assessment approach warrant the creation of a new assessment tool that enables performance feedback for improving a student's performance and/or understanding of the skill(s) being assessed [6,22]. Performance feedback needs to accommodate a developmental approach to learning and integrating a process-oriented assessment [7,18,23]. To address these limitations, the Refuse, Assisted, Effective, Efficient (RAEE) assessment tool was developed by the authors (Table 1) [24,25] and the face [25] and content [24] validity confirmed. The RAEE is a tool incorporating a seven-point Likert scale that differentiates between levels of performance of a skill being demonstrated from ‘outright refusal to attempt a task’ to ‘being efficient while completing a task’. The seven points are divided into four subheadings (represented by its name) and utilise a ‘stop light’ schematic to highlight the hierarchical nature of the assessment criteria.

**Table 1:** Assessment Tool – RAEE (Refuse, Assisted, Effective, Efficient. The RAEE is designed to apply to movement skills, water safety knowledge and understanding with the rating system broadly broken into four main areas: Refusal – Assisted – Effective – Efficient.

1	Refused to attempt task.	
2	Attempted task – did not complete.	
3	Attempted task – completed with assistance.	Assistance is defined as kickboards, noodles, floats or physical assistance from another person.
		Breathing is controlled throughout task – i.e. no choking, gasping or reliance on aids to breathe comfortably.
4	Completed task with **difficulty**, little or no confidence and/or inefficiently.	**Difficulty** is defined as the inability to coordinate the individual components of the stroke as a whole (i.e. body position, arm action, leg action and breathing).
5	Completed task with **moderate ease** and lacking**confidence** and/or efficiency (correct stroke technique as described in the AUSTSWIM manual).	**Moderate ease** is defined as the ability to coordinate the individual components of the stroke as a whole, but not having the ability to maintain that coordination over distances greater than 10 ​m.
		**Confidence** is demonstrated through non-reliance on aids such as goggles, pool edge, shallow water and/or lack of anxiety in the water.
6	Completed task with **ease**, but lacking confidence and/or efficiency (correct stroke technique as described in the AUSTSWIM manual).	**Ease** is defined as the ability to coordinate the individual components of the stroke as a whole and maintaining that coordination over distances greater than 10 ​m.
7	Completed task with ease, confidence and with moderate or high **efficiency** (correct stroke technique as described in the AUSTSWIM manual).	**Efficiency** is demonstrated through reduction of frontal and eddy resistance affecting the body while moving through water – i.e. body position, maintaining streamline, reducing time/distance from streamline position.

Therefore, the aim of this study is to investigate both the inter- and intra-rater reliability of assessments using two different assessment approaches of water safety skills. It is hypothesised that use of the RAEE assessment tool will demonstrate higher inter- and intra-rater reliability when compared to use of the C/NYC assessment method across five water safety skills sourced from the AWSC's benchmark.

## 2 Method

### 2.1 Participants

Twelve participants (raters) from a single Gold Level AUSTSWIM recognised swim centre (exclusively employing AUSTSWIM trained instructors) in regional New South Wales, volunteered to participate in this study and provided written and informed consent. All raters were currently licensed AUSTSWIM swimming and water safety teachers with teaching experience categorised as either novice (<2 years' experience, n ​= ​1) or experienced (>2 years’ experience, n ​= ​9). All participants had participated in recent in-service training at this single swim centre that was intended to standardise the performance and assessment criteria for swimming and water safety skills taught at the swim school. This cross-sectional study design research was approved by the Human Research Ethics Committee (H-2016-0224).

### 2.2 Video data set

All assessments were made on videos of competent adult volunteers (n ​= ​28; 18–34 years, performing the skills. Fourteen individual skills were chosen from the AWSC benchmark, RLSSA (2016 version) [9], with each swim task demonstrated to the award guided distance, time and/or depth. These skills were performed in either indoor 25-m or outdoor 50-m heated pool as a continuous sequence, videoed (Elite Wi-Fi action camera HN-TOP1, Neos, China), and edited to create filmed clips of ∼10 ​s. The benchmark skills included were: Crouch dive (n ​= ​15); Feet first scull (n ​= ​18); Sidestroke (n ​= ​19); Compact jump (n ​= ​16); and Clothed Survival Swim (n ​= ​8), and the rationale for the skills included are outlined in the Supplementary. A total of 76 benchmark skill video clips were allocated a numerical code and randomised using a random number generator.

### 2.3 Procedures

Three rating sessions a minimum of one month apart were held, where in a group setting, raters viewed and individually rated the randomised video data sets of the 76 recorded benchmark tasks. In session 1 (S1), raters were provided with verbal instructions on how the research was to be conducted, explanation on how to use the C/NYC, RAEE and the rating recording sheets. Independence between raters was not maintained in S1 where there was collaboration between group members, this was rectified in latter sessions. Raters viewed the video clips (projected onto a wall/screen via video projector) and rated each video clip or task as C/NYC. The raters then re-watched the same video clip, rated the task using the RAEE scale of 1–7. This procedure was repeated for all 76 tasks of the video data set. The second session (S2) and third session (S3), the viewing and rating of the randomised video clips was repeated. Raters unable to attend the any session(s), were provided with a USB (pre-populated with the randomised video data sets) and an assessment sheet to conduct the ratings of the task independently.

### 2.4 Statistical analysis

Repeated measures analysis was used to calculate the inter- and intra-rater reliability, with weighted kappa utilised for inter-rater analysis and Chi squares for the intra-rater analysis. Descriptive analysis and analytics of rating assessment tool data sets (C/NYC binary data; RAEE ordinal data) was completed. Data for raters who only completed S1 were removed (n ​= ​2). The percentage agreement between raters for each task within each session and for each task over the three sessions was calculated (JMP Pro 14, Attribute Gauge Chart, SAS Institute Inc., Australia).

The kappa coefficient measures true agreement, beyond that of chance, although does not differentiate between random and systematic disagreement, which needed to be taken into account [26]. Fleiss's Multi-rater Kappa can identify disagreement (weighted kappa) and was used to calculate inter-rater reliability using IBM SPSS Statistics 26 (IBM, Chicago, Illinois) [26,27]. Intra-rater reliability was conducted for all three sessions using the crosstabs kappa function (chi squares) of IBM SPSS 25 with 95 ​% confidence interval calculated (JMP Pro 14, Attribute Gauge chart). Intra-rater reliability is reported as an average kappa across raters for each task, S1vS2, S2vS3, and S1vS3. Strength of agreement (kappa) interpretation of reliability was categorised as: ≤0.1 poor, 0.1–0.2 slight, 0.21–0.4 fair, 0.41–0.6 moderate, 0.61–0.8 substantial, and 0.81–1.0 almost perfect [28,29].

## 3 Results

### 3.1 Inter-rater reliability - percentage agreement

Percentage agreement is reported in Table 2. Higher percentage agreement was observed in the C/NYC (range: 48 ​% S1, sidestroke to 76 ​% S3, clothed survival swim) compared to the RAEE (range: 18 ​% S2 and S3, crouch dive to 29 ​% S3, sidestroke and clothed survival swim).

**Table 2:** Percentage Agreement across raters/session by task.

Session	Crouch Dive n ​= ​15		Feet first scull n ​= ​19		Sidestroke n ​= ​18		Compact jump n ​= ​16		Clothed survival swim n ​= ​8	
	%	SD	%	SD	%	SD	%	SD	%	SD
Competent/Not yet competent assessment method										
1	72	5	58	4	48	6	52	8	74	1
2	61	6	58	4	53	1	65	4	74	0
3	68	1	70	8	60	6	64	4	76	1
Average	67	4	62	5	53	4	60	6	74	0
Refuse, assisted, effective, efficient assessment tool										
1	24	4	24	0	25	1	18	1	27	1
2	18	2	24	1	26	1	22	2	27	0
3	18	2	25	1	29	2	19	0	29	1
Average	20	3	24	1	26	1	20	1	28	1

### 3.2 Inter-rater reliability – Fleiss's multi-rater kappa

**Fig. 1:**
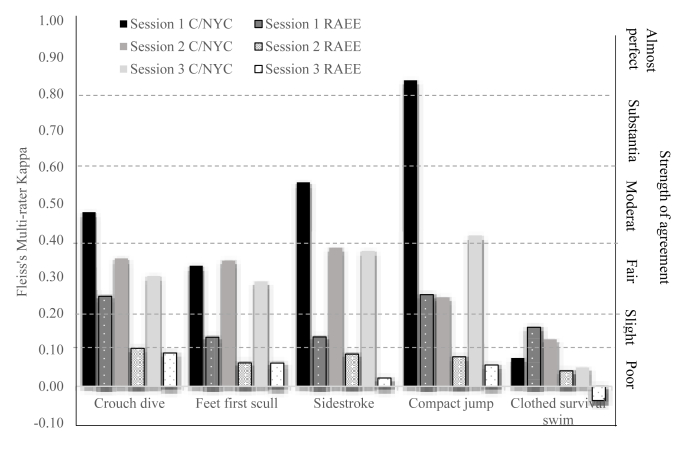
Strength of agreement using different assessment method/tool: C/NYC vs. RAEE for demonstrated skills across three sessions.

Strength of agreement for both methods (Fig. 1), showed S1 had almost perfect agreement for the compact jump using C/NYC. Level of agreement across all skills was higher (fair/moderate) using the C/NYC compared to the RAEE (poor/fair). The clothed survival task was the only outlier, in which the RAEE (S1) and C/NYC (S2) showed slight agreement, and all other sessions showed poor agreement. Moderate agreement was present using C/NYC for the crouch dive and sidestroke (S1) and compact jump (S3). A fair level of agreement was found using C/NYC for feet first scull (S1), crouch dive, feet first scull, sidestroke and compact jump (S2) and crouch dive, feet first scull and sidestroke (S3). The RAEE only observed fair level of agreement for the crouch dive and compact jump (S1). For the remainder of tasks and sessions, the RAEE observed only a slight level of agreement for the feet first scull and sidestroke (S1), for all tasks (S2) and for the crouch dive, feet first scull, sidestroke and compact jump (S3).

### 3.3 Intra-rater reliability

Table 3 compares the average (across all raters) strength of agreement between sessions. The C/NYC demonstrates the highest level of agreement (moderate) between all three session comparisons for the crouch dive and clothed survival swim; between two comparisons for sidestroke (S1vS2 and S2vS3) and compact jump (S2vS3 and S1vS3); and once for feet first scull (2v3). A fair level of agreement was found for sidestroke (S1vS3), compact jump (S1vS2) and for two comparisons for feet first scull (S1vS2 and S1vS3).

**Table 3:** Averages of Intra-rater reliability and 95 ​% Confidence Interval between sessions.

Competent/Not yet Competent assessment method															
Between Sessions	Crouch dive			Feet first scull			Sidestroke			Compact jump			Clothed survival swim		
	kappa	Lower 95 ​% CI	Upper 95 ​% CI	kappa	Lower 95 ​% CI	Upper 95 ​% CI	kappa	Lower 95 ​% CI	Upper 95 ​% CI	kappa	Lower 95 ​% CI	Upper 95 ​% CI	kappa	Lower 95 ​% CI	Upper 95 ​% CI
1 vs 2	0.59	0.35	0.82	0.31	0.05	0.58	0.42	0.22	0.61	0.38	0.28	0.48	0.46	0.52	0.86
2 vs 3	0.56	0.42	0.69	0.41	0.23	0.59	0.45	0.27	0.62	0.43	0.27	0.58	0.52	0.12	0.92
1 vs 3	0.44	0.22	0.66	0.31	0.11	0.50	0.40	0.28	0.52	0.52	0.34	0.70	0.50	−0.02	1.02
Refuse, assist, effective, efficient assessment tool															
1 vs 2	0.23	0.10	0.35	0.17	0.03	0.31	0.11	−0.02	0.25	0.14	0.04	0.24	0.16	0.01	0.30
2 vs 3	0.27	0.18	0.35	0.14	0.05	0.23	0.21	0.09	0.33	0.29	0.19	0.39	0.23	0.09	0.36
1 vs 3	0.18	0.01	0.34	0.07	−0.06	0.21	0.11	−0.02	0.24	0.14	0.02	0.25	0.06	−0.04	0.18

The highest level of agreement (fair) for the RAEE assessment tool was observed in the crouch dive (S1vS2, S2vS3) and sidestroke, compact jump and clothed survival swim (S2vS3). A slight level of agreement was found for feet first scull (S1vS2, S2v3S); sidestroke and compact jump (S1vS2, S1vS3); and for clothed survival swim (S1vS2). The lowest level of agreement (poor) was found for both feet first scull and clothed survival swim (S1v3S).

**Fig. 2:**
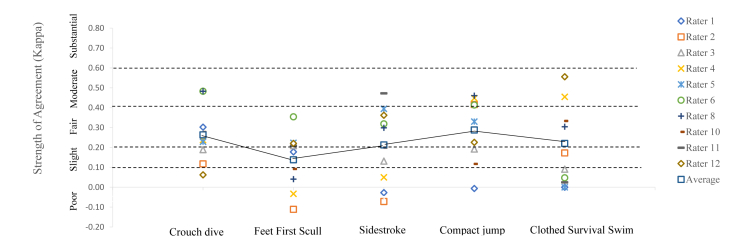
Intra-rater reliability: Individual rater strength of agreement for demonstrated tasks using RAEE assessment tool – between sessions 2 and 3.

The clothed survival swim had the greatest range of 95%CI using the C/NYC (S2vS3; 0.12–0.92) and RAEE (S1vS2; 0.01–0.30. The smallest range for 95%CI using C/NYC was for the compact jump (S1vS2; 0.28–0.48) and using the RAEE was for the crouch dive (S2v3S; 0.18–0.35). Figure 2 shows the individual ratings for the ten raters with the average highlighted by the trend line. The comparison between S2 and S3 visually displays the variability (95%CI) of each individual rater across both sessions and tasks.

## 4 Discussion

It remains unknown if the AWSC's benchmark of the minimum standard of swimming, water safety and self-rescue skills to be achieved by all Australian children in learn-to-swim programs [8,9,30,31] are being met by the learn-to-swim industry due to the known issues with validity and reliability of assessments [6,18,19]. This study explored the inter- and intra-rater reliability of two approaches to assessing water safety skills: the current default C/NYC assessment method and the new RAEE assessment method. The study's hypothesis was not supported, there was limited/poor inter and intra rater reliability for both methods with the RAEE method demonstrating lower inter- and intra-rater reliability than the C/NYC method across five water safety skills. The similar results of the reliability of these two methods reflects the lack of accuracy in the measurement of aquatic skills within learn-to-swim assessments. There was evidence that confounding variables could have influenced the reliability of the assessments within this study such as: the lack of clear, criterion referenced standards; bias created by rater knowledge, experience and/or perceptions of pedagogical approach applied to assessment; rater knowledge of and application of specific skill criteria, greater number of options to choose from when assessing using the RAEE compared to the C/NYC, and a lack of terminology that enables clear communication of assessment outcomes.

Inconsistency of rater responses for inter- and intra-rater agreement and percentage agreement regardless of which assessment approach was used, suggests a lack of familiarity with the standards or ‘must see’ criteria set by the assessment guide for each skill. This was despite that all participants attending the same in-service training at the swim centre prior to completing the rating that was intended to standardise the performance and assessment criteria for swimming and water safety skills taught at the swim school. The average percentage agreement across all three sessions was higher for C/NYC (53 ​%[±4] to 74 ​%[±0]) and RAEE (20 ​%[±3] to 28 ​%[±1]) across all skills yet resulted in slight or poor strength of agreement (weighted kappa). While the overall percentage agreement for C/NYC was higher than the RAEE across all skills, the average percentage agreement for the crouch dive, feet first scull, sidestroke and compact jump ranged between 53 ​%(±4) to 67 ​%(±4), which is not indicative of strong inter-rater agreement (≥80 ​%) [32].

Reliably assessing motor skills such can be difficult due to differences in rater experience, knowledge of standards and/or assessment tool(s); the number of raters utilised; the skill focus; and the use of field-based settings [[33], [34], [35]]. The assessment focus can differ depending on whether raters are looking for evidence *of* learning (the ability to demonstrate skill) or *for* learning (the ability to demonstrate appropriate application of skill) [36,37]. Often this equates to whether the raters's focus is on the outcome of learning and whether they can perform the skill or not (product oriented) or the process of skill acquisition and how they are able to perform. Adopting a process focus has been shown to improve the rater's ability to identify and correct movement deficiencies more easily and can influence reliability of the rater's assessment [22,34,36].

A lack of agreement was evident between raters across all tasks and increased over time. The strength of agreement decreased across sessions for three of the skills (crouch dive, sidestroke, compact jump). The greater strength of agreement from the S1 could be attributed to the collaborative session approach, as raters discussed the videos and could therefore be influenced by each other in deeming the skill demonstrated C/NYC [26]. The S2 and S2 assessments had no discussion/collaboration between raters and were completed independently of the group sessions (S2 40 ​% independent ratings, S3 80 ​% independent ratings).

The paradoxical presentation of high agreement with a low kappa is indicative of the existence of a prevalence effect and bias [38]; a finding observed for the clothed survival swim. For example, a rater declaring they believe a key performance indicator of the compact jump is that the airway should be protected (covering mouth and nose with hand) (prevalence) and expecting that action to be observed (bias). When raters are assessing C/NYC and there is a difference in the proportion of agreements, a prevalence effect exists. This makes interpreting the magnitude of agreement (kappa) difficult if the prevalence index is not taken into account [26]. Bias is the extent that raters disagree on the proportion of positive (or negative) assessments and similar to the prevalence index, the bias index should also be taken into account when interpreting the kappa [26]. In this study, raters did not declare or record their beliefs or bias prior to assessment, therefore, neither the prevalence nor the bias indexes could be calculated.

Interpreting the strength of agreement is more difficult for ordinal scale tools as prevalence and bias cannot be attributed in the same manner as to a dichotomous scale [39]. Dichotomous and three point scales are traditionally associated with a lower inter-rater reliability when compared to scales between four and seven points, as a result of the limited range for disagreement [40]; the opposite effect was demonstrated in this study. The RAEE strongest level of agreement was fair (crouch dive, compact jump) with all other tasks reaching slight (sidestroke, feet first scull) or poor (clothed survival swim), and by the S3 all the tasks had a poor level of agreement between and within raters. This could be indicative of low knowledge and understanding of the performance indicators for each of the skills and a limited understanding of how to use the rating scale. The combination of these factors and the RAEE being ordinal that would therefore be more sensitive to disagreement, may be responsible for its low inter-rater reliability observed.

A combination of improved knowledge and understanding of the underpinning principles of assessment and clear communication prior to assessment can only improve inter-rater reliability [[24], [25], [26],^34^]. The overall low inter-rater reliability for both methods in this study, highlights this need for improved training for instructors; a finding reaffirmed by our face [25] and content [24] validity RAEE studies. Instructors need to understand the factors impacting on assessment in key areas regarding methods, tools and techniques [41]. They need to be able to choose (and communicate) which assessment approach is the most appropriate for any given situation (product, process or combined) [33,42,43]. The ability to improve inter-rater reliability is predicated on the instructors being able to accurately identify what skills are being taught (content); where they are being taught (environment(s)); how they are taught (error correction or developmental) and why (the philosophical goals or intent of the program/swim school/instructor - competitive, survival and/or recreational) prior to instruction in swimming and water safety commencing. Instructors need to augment their understanding of the skill components and key performance indicators incorporated in current teaching practises would allow them to identify the presence or absence of a specific skill component (prevalence if using C/NYC); and identify what (if any) bias (expectation of a specific skill component to be present or absent) [44].

The low knowledge and understanding of underpinning assessment principals and lack of clear communication that appear to be responsible for the anomalies in inter-rater reliability, would similarly affect the low intra-reliability observed within this study. Moderate agreement for C/NYC was observed across all skills. There are several limitations in the statistical package available (SPSS) that restrict the ability to calculate weighted kappa over multiple sessions and skills [45]. Firstly, the S1 and S2 differences need to be excluded due to lack of independence. Secondly, caution is needed when comparing the magnitude of kappa measured on dissimilar scales (dichotomous C/NYC v ordinal RAEE), with unweighted kappa not suitable for ordinal scales [26]. In order to measure the actual strength of agreement for each rater across different skills, a different repeated measure study design would need to be incorporated allowing the calculation for weighted kappa [45]. The sensitivity of the ordinal scale, lack of familiarity with a process orientated assessment tool and lack of clear skill definitions combined with low knowledge of how core proficiencies are assessed, impact upon the observed intra-rater reliability.

### 4.1 Limitations

The limited sample size of raters (n ​= ​10), from a single swim school cannot be extrapolated to other swim schools, a larger sample with a wider demographic group, training background and from different swim schools should be considered for future studies. The use of a group setting may have impacted the independence between raters and may have artificially increased the strength of agreement be due to: unfamiliarity with the process of completing the tasks of the research study (i.e. fear of making mistakes); strongly held opinions voiced by individual raters and exploration of other rater's perception of the ‘correct’ interpretation of the standards. A familiarisation session, before the rating of the assessment tasks are recorded, is encouraged in future studies to mitigate this effect. The videos used for the assessment tasks only featured competent swimmers who willingly volunteered to be videoed while demonstrating the swimming tasks, indicating a volunteer bias and a limited the range of aquatic competencies. Statistical software limitations of the SPSS software meant that the weighted kappa could not be used to calculate the intra-rater reliability [45] and the kappa score report that is lower than weighted kappa as the strength of disagreement is not treated differently [46].

## 5 Conclusion

Irrespective of the assessment approach taken, the inter- and intra-rater reliability was limited when assessing water safety skills. There appears to be a general lack of consensus relating to what constitutes proficiency in performing aquatic skills and particularly those related to water safety, despite all participants attending the same in-service aquatic training centre prior to completing the rating. The raters lacked a clear understanding of the complexities involved in assessment, including the requirement for a foundation of what it looks like to perform each of these skills well. Taking measures to improve reliability within the aquatics industry could improve the capacity to accurately measure the impact of swimming and water safety instruction as a drowning prevention tool.

## Declaration of competing interest

The authors declare the following financial interests/personal relationships which may be considered as potential competing interests: Suzi Edwards, Carole James and Michela Bruton has no interest to declare. Nina Nyitrai is a AUSTSWIM Trainer Assessor, a contract trainer for Royal Life Saving Society Australia and is the owner of Swim4Surivial that provides in-service training and education for swimming pool and swim school centres.
